# The risk of dengue for non-immune foreign visitors to the 2016 summer olympic games in Rio de Janeiro, Brazil

**DOI:** 10.1186/s12879-016-1517-z

**Published:** 2016-04-29

**Authors:** Raphael Ximenes, Marcos Amaku, Luis Fernandez Lopez, Francisco Antonio Bezerra Coutinho, Marcelo Nascimento Burattini, David Greenhalgh, Annelies Wilder-Smith, Claudio José Struchiner, Eduardo Massad

**Affiliations:** School of Medicine, University of São Paulo and LIM01-HCFMUSP, São Paulo, SP Brazil; Center for Internet Augmented Research &Assessment, Florida International University, Miami, FL USA; Escola Paulista de Medicina, Universidade Federal de São Paulo, São Paulo, SP, Brazil; University of Strathclyde, Glasgow, UK; Lee Kong Chian School of Medicine, Nanyang University, Singapore, Singapore; Programme of Scientific Computation, Fundação Oswaldo Cruz, Rio de Janeiro, Brazil; London School of Hygiene and Tropical Medicine, London, UK

**Keywords:** Dengue, Mathematical models, Risk assessment, Olympic games, Travel medicine

## Abstract

**Background:**

Rio de Janeiro in Brazil will host the Summer Olympic Games in 2016. About 400,000 non-immune foreign tourists are expected to attend the games. As Brazil is the country with the highest number of dengue cases worldwide, concern about the risk of dengue for travelers is justified.

**Methods:**

A mathematical model to calculate the risk of developing dengue for foreign tourists attending the Olympic Games in Rio de Janeiro in 2016 is proposed. A system of differential equation models the spread of dengue amongst the resident population and a stochastic approximation is used to assess the risk to tourists. Historical reported dengue time series in Rio de Janeiro for the years 2000-2015 is used to find out the time dependent force of infection, which is then used to estimate the potential risks to a large tourist cohort. The worst outbreak of dengue occurred in 2012 and this and the other years in the history of Dengue in Rio are used to discuss potential risks to tourists amongst visitors to the forthcoming Rio Olympics.

**Results:**

The individual risk to be infected by dengue is very much dependent on the ratio asymptomatic/symptomatic considered but independently of this the worst month of August in the period studied in terms of dengue transmission, occurred in 2007.

**Conclusions:**

If dengue returns in 2016 with the pattern observed in the worst month of August in history (2007), the expected number of symptomatic and asymptomatic dengue cases among tourists will be 23 and 206 cases, respectively. This worst case scenario would have an incidence of 5.75 (symptomatic) and 51.5 (asymptomatic) per 100,000 individuals.

## Background

Brazil will host the Summer Olympic Games, which will take place mainly in Rio de Janeiro, to be held between August 5–21, 2016, followed by the summer Paralympics, between 7–18 September. (http://www.rio2016.com/en). The Olympics is expected to attract some 400,000 foreign visitors in addition to about 600,000 domestic supporters from other states of Brazil. Mass gatherings such as the Olympics are of particular public health concern as infectious diseases can rapidly affect large numbers of persons [1]. Dengue is a viral infection caused by 4 dengue serotypes (DENV 1–4) transmitted by mosquitoes that is an increasing problem in Brazil and other countries in the tropics and subtropics [2]. As Brazil is the country with the highest number of dengue cases worldwide [3, 4], concern about the risk of dengue for travelers is justified. Indeed, dengue was the most frequent cause of systemic febrile illness in travelers to Brazil, reported in almost 6 % of returning travelers with any illness from Brazil as documented by GeoSentinel, a global network of travel medicine providers [5]. Hence, the FIFA World Football Cup that took place in Brazil in 2014 motivated extensive scientific and media communication around the potential risk of dengue to foreign visitors [6–12]. One modelling study predicted the risk to foreign visitors to be as high as 33 (varying from 3 to 59) in 607,051 attendees at the football games [7]. Fortunately, the year 2014 was a year of unusually low dengue incidence in all the cities hosting the games, and in the end only 3 cases were documented by the Brazilian authorities, one in a Japanese citizen and 2 in US citizens [13].

Dengue outbreaks shows a strong periodical pattern within a year because of the influence of temperature and precipitation on mosquito abundance and vectorial capacity [14, 15] and also because a threshold in the proportion of susceptible may be reached [ArXhiV: submitted]. Dengue epidemics are often difficult to predict but easy to understand. Epidemic dengue transmission differs from year to year, often presenting a cyclical pattern. A year with high dengue activity is often followed by a year with lower incidence due to herd immunity. In adition, the interaction between climate and dengue activity is not linear, because population density, socio-economic conditions, sanitation and people mobility also play major roles in dengue epidemiology. There are also genotypic changes and introduction of new sero-and genotypes [16].

**Fig. 1:**
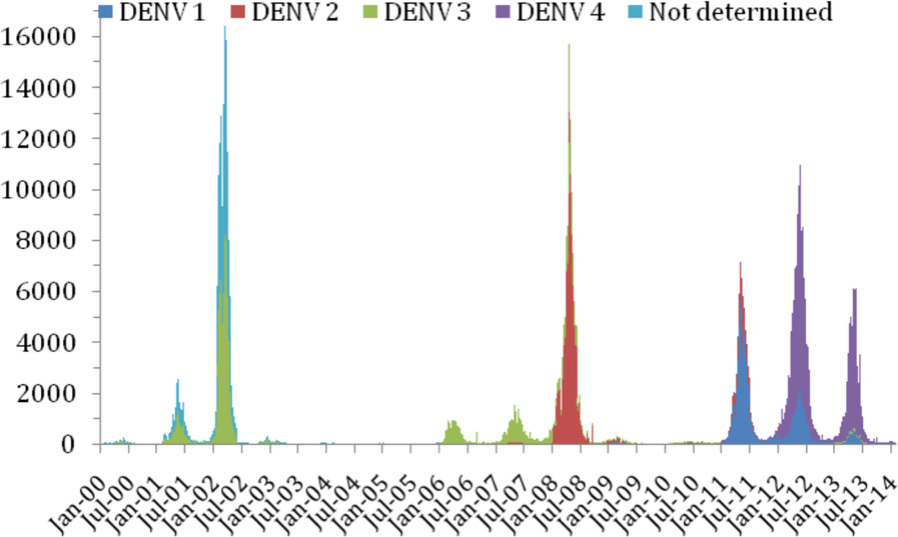
Number of reported cases of dengue in Rio de Janeiro in the period January 2000-July 2014. Data from SINAN (the Brazilian national notifiable diseases system)

Dengue virus (DENV) was reintroduced into Brazil in 1981 and by 1995 it had spread throughout the country [3, 4]. Dengue cases, and many other compulsory notifiable infectious diseases, are weekly reported to the official reporting system of the Brazilian Ministry of Health (called SINAN) database. More than 7 million dengue cases have been reported in Brazil, and Brazil is now the country with the highest dengue incidence in the world [17]. By 2007 the number of dengue hemorrhagic fever (DHF) cases had more than doubled [18]. The three dengue serotypes, DENV-1, DENV-2, DENV-3 haven been circulating widely in Brazil for years, but in 2011 DENV-4 invaded the country and in 2012 the highest incidence was reported to date, 95 % of which were due to DENV-1 and DENV-4 (dtr2004.saude.gov.br/sinanweb/tabnet/dh?sinannet/dengue/bases/denguebrnet.def). Figure 1 shows the year-to-year oscillations of dengue outbreaks.

**Fig. 2:**
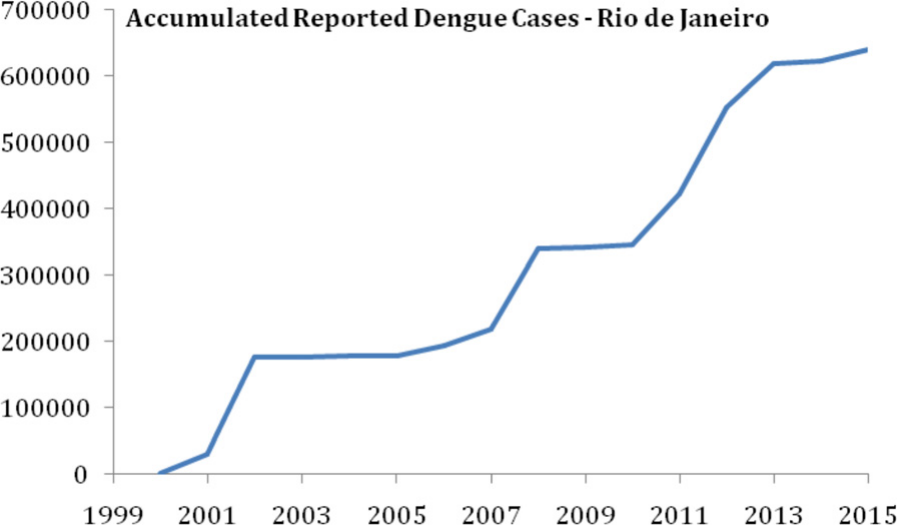
Accumulated number of reported dengue cases in Rio de Janeiro, 2000-2015. We assume that the segments of the curve that show no increase in the cases are cases where a threshold of susceptible was reached. Note also that there are changes in the slope of the curve that indicate the epidemics propagating through the city. Finally the number of accumulated cases in the last outbreak twice the number of the previous ones. This indicates two virus circulating without competition. This matter is analyzed in [arXiv: submitted]

Figure 2 shows the number of accumulated dengue cases in the period between 2000 and 2015 in Rio, corresponding to the data observed in Fig. 1.

This figure shows several features that will be analyzed in another paper. From it, we see that DENV-3 started in 2000 and circulated until 2007. In 2008, until 2010, DENV-2 predominated. From 2011 until 2015, two serotypes, DENV-1 and DENV-4 started circulating. Note that the number of cases in the last outbreaks is about twice as high as the number of cases observed in previous outbreaks. This strongly suggests that the two serotypes do not compete against each other.

Rio de Janeiro, the venue for the 2016 Olympic Games, has been of major importance for the epidemiology of dengue in Brazil. After the DENV 1–4 introductions in 1986, 1990, 2000 and 2011, respectively, the city has suffered explosive outbreaks [19].

Properly quantifying the risk of dengue for foreign visitors to the Olympics is important. On one hand, we want to avoid the overly alarming news and overestimates of dengue that circulated before the 2014 FIFA World Cup in Brazil [6–9], on the other hand, we want to be adequately prepared for the Summer Olympics. In this paper we propose to estimate the risk of dengue for foreign visitors attending the summer Olympics in Rio de Janeiro in 2016, in the period between the 30th and 32nd epidemiological week of the year. We also discuss the strength and limitations of the model taking into account the experiences with the models proposed for estimating the risk of dengue during the FIFA World Cup in 2014.

## Methods

We propose a stochastic model for the calculation of the per capita probability of dengue infection for visitors to Rio de Janeiro in period between the 30th and the 32nd epidemiological week of the year 2016. The model is based on the calculated force of infection, averaged over the entire city for each years since the year 2000. The risk of dengue during the 3 first weeks of the months of August of each year of the historical series analyzed was then calculated.

### The Model for dengue among the local inhabitants of Rio de Janeiro

The model assumes that the population of humans and mosquitoes are subdivided into: susceptible humans *S*_*H*_, infected humans *I*_*H*_, recovered (and immune) humans *R*_*H*_, total humans *N*_*H*_, susceptible mosquitoes *S*_*V*_, infected and latent mosquitoes *L*_*V*_, and infected and infectious mosquitoes *I*_*V*_. The dynamics of an ideal model is described by the set of equations shown in Table 1 [20].

**Table 1:** Model describing dengue dynamics

$$ \begin{array}{l}\frac{d{S}_H}{dt}=- ab{I}_V\frac{S_H}{N_H}-\mu {S}_H+\mu {N}_H,\\ {}\frac{d{I}_H}{dt}= ab{I}_V\frac{S_H}{N_H}-\left(\mu +\gamma +\alpha \right){I}_H,\\ {}\frac{d{R}_H}{dt}=\gamma {I}_H-\mu {R}_H\\ {}\frac{d{S}_V}{dt}=-ac{S}_V\frac{I_H}{N_H}+{\mu}_V\left({N}_V-{S}_V\right),\\ {}\frac{d{L}_V}{dt}=ac{S}_V\frac{I_H}{N_H}-{\mu}_V{L}_V-{\gamma}_V{L}_V,\\ {}\frac{d{I}_V}{dt}={\gamma}_V{L}_V-{\mu}_V{I}_V,\\ {}{N}_H={S}_H+{I}_H+{R}_H,\end{array} $$ (1)	

In Table 1, *a* is the daily biting rate of the mosquitoes (that is, the average number of bites each mosquito inflicts in the human hosts), *b* is the proportion (that is, a probability) of infected bites that are actually infective for humans, *c* is the proportion (that is, a probability) of bites that are due to infective mosquitoes, *μ* is the mortality rate of humans, *γ* is daily the recovery rate of humans, *μ*_*V*_ is the daily mortality rate of mosquitoes, 1/*γ*_*V*_ is the average latency period in days in the mosquitoes and *α* is the dengue induced daily mortality rate of humans. The quantities *S*_*H*_, *I*_*H*_, *R*_*H*_, etc., are densities [21] and although very simple, this model can reproduce most of the characteristics of dengue outbreaks among visitors [22].

### Calculating the per capita probability of infection

Assuming a closed population of size *N* in the absence of competitive risks, the risk of infection can be stochastically calculated by a two state model without recovery. Such a Susceptible-Infected (SI) model is one in which *S* individuals are susceptible to the infection and *I* individuals are those who acquired the infection, remaining infected for life. This is an approximation to the dengue reality and it is justified by the fact that visitors are in relatively low number with respect with the local population and they spend a relatively short period of time in the visited place. Visitors, therefore, are assumed to be subject to the local force of infection but do not contribute to it. The consequence of this assumption is that we can use a linear model, which simplifies the calculation of the risk of infection. The details of the risk calculation are shown in Table 2.

**Table 2:** A stochastic SI approximation to the risk of dengue for visitors

Let us start by calculating the probability that *x* individuals are in the state *S* and *y* individuals are in the state *I* at time *t + Δt*: *P*_*x*,*y*_(*t* + *Δt*) = *P*_*x*,*y*_(*t*)(1 − *λxΔt*) + *P*_*x* + 1,*y* − 1_ *λ*(*x* + 1)*Δt* (2) In equation (2) the first term refers to the probability that there were *x* and *y* individuals at time *t* in the states *S* and *I* respectively, and that no susceptible individuals *x* acquired the infection in the period. The second term refers to the probability that there were (*x* + 1) and (*y*-1) individuals at time *t* in the states *S* and *I* respectively, and that one susceptible individual acquired the infection in the period. From equation (2) it follows that: ${\scriptscriptstyle \frac{dPx,y(t)}{dt}}=-\lambda x{P}_{x,y}(t)+\lambda \left(x+1\right){P}_{x+1,y-1}(t).$ (3) The general expression for the Probability Generation Function (PGF), *G*(*u*,*v*,*t*), is given by: *G*(*u*, *v*, *t*) = ∑_*y* = 0_^*N*^ *u* ^*x*^ *v* ^*y*^ *P*_*x*,*y*_(*t*). (4) For the particular model expressed in equation (4) it is possible to deduce that the PGF is: *G*(*u*, *v*, *t*) = [(*u* − *v*)*e* ^− *λt*^ + *v*]^*N*^.. (5) Now the average number of infected individuals, *y*, at time *t* can be calculated by taking the first partial derivative of the PGF with respect to *v* at *u*,*v* = 1: ${\scriptscriptstyle \frac{\partial G\left(u,v,t\right)}{\partial v}}\left|{}_{u,v=1}=N\left(1-{e}^{-\lambda t}\right)\right..$ (6) Hence, the average per capita risk of infection, *π* is given by: *π* = 1 − *e* ^− *λt*^. (7) The variance of the probability distribution for the number of infected individuals at time *t* is given by: $var\left[y\right]={\scriptscriptstyle \frac{\partial^2G\left(u,v,t\right)}{\partial {v}^2}}\left|{}_{u,v=1}\right.+{\scriptscriptstyle \frac{\partial G\left(u,v,t\right)}{\partial v}}{\left|{}_{u,v=1}-\left[{\scriptscriptstyle \frac{\partial G\left(u,v,t\right)}{\partial v}}\left|{}_{u,v=1}\right.\right]\right.}^2$ (8) which results in: *var*[*y*] = *Ne* ^− *λt*^[1 − *e* ^− *λt*^]. (9)	

In a model such as the one shown in Table 2, that excludes any competitive risks (that is, neither recovery/death before returning home nor returning home before recovering from the infection), the PGF (5) is the PGF of a Binomial Distribution (susceptibles either acquire the infection or not) with *N* trials and probability of success *π* given by (7) and variance given by (9).

The force of infection of dengue, however, shows a marked oscillation with a peak in the end of the summer, decreasing thereafter. Therefore, to determine the risk of dengue for visitors in the period of the Olympic Games (August 2016) it is necessary to take this into account, and equation (7) now becomes equation (10), shown in Table 3:

**Table 3:** The risk of dengue for visitors taking into account the observed oscillation in transmission

$$ \pi \left(\omega, \varOmega \right)=1- exp\left[-{\displaystyle \underset{\omega }{\overset{\omega +\varOmega }{\int }}\lambda (s)ds}\right]. $$ (10)	

In Table 3, tourists are assumed to arrive at time *ω*, remaining in Rio until time *ω* + *Ω*, when they leave.

Note that the dynamics of dengue are actually given by the set of equations (1) which is a host-vector model in which the epidemiological dynamics of the human population is of Susceptible-Infected-Removed (SIR) type. However the timescale on which the epidemiological changes occur is typically very long compared with the short mosquito lifecycle.

The probability that each visitor develops dengue, therefore, is given by equation (10) of Table 3. Thus the number of dengue cases amongst tourists is again Binomial with parameters *N* and *p* = *π*(*ω*,*Ω*).

So we need to find *λ*(*t*), to finally calculate the risk value for visitors. Therefore, we rewrite the first two equations of system (1), as shown by system (11) in Table 4.

**Table 4:** SI approximation of dengue transmission among visitors

$$ \begin{array}{l}\frac{d{S}_H}{dt}=-\lambda (t){S}_H-\mu {S}_H,\\ {}\frac{d{I}_H}{dt}=\lambda (t){S}_H-\left(\mu +\gamma +\alpha \right){I}_H.\end{array} $$ (11)	

In Table 4, the force of infection *λ*(*t*) = *abI*_*V*_/*N*_*H*_ is the rate of attack of a single susceptible individual at time *t* and *λ*(*t*)*S(t)* is the dengue incidence (SINAN data) new cases per time unit.

### Estimating the Force of Infection of Dengue in Rio de Janeiro

In this section we show how to estimate the force of infection of dengue in Rio de Janeiro. We assume that visitors attending the Olympic Games are subject to the same force of infection of dengue as the local residents. This is a strong assumption and it is based on the observation that, on the one hand, some of the tourists tend to be hosted in hotels with air conditioning and therefore with less contact with the *Aedes aegypti* mosquitoes. On the other hand, a growing number of tourists prefer to stay in the shanty towns of Rio, subject to high exposure to the mosquitoes. Hence, on average, tourists are expected to receive the same number of potentially infective bites as local residents. The result of this assumption is an upper bound of the expected risk to visitors.

The force of infection of dengue in Rio de Janeiro shows a marked oscillatory behavior with cases starting to grow early in January, peaking in late April, and declining thereafter. By considering the notified number of weekly cases reported to the SINAN database from 2000 until 2015, we can calculate the time-dependent force of infection of dengue year by year in this period of 16 years.

To estimate the force of infection of dengue in Rio in the years analyzed, we modeled the time-dependent force of infection as a function with the “corrected Gaussian” form, as shown in Table 5:

**Table 5:** Dengue force of infection modeled as a Gaussian function

$\lambda (t)={c}_1 exp\left[-\frac{{\left(t-{c}_2\right)}^2}{c_3}\right]F(t),$ (12) where *c*_1_ is a scale parameter that determines the maximum incidence, *c*_2_ is the time at which the maximum incidence is reached, *c*_3_ represents the width of the time-dependent incidence function and *F*(*t*) is an ad hoc function introduced to both improve the model fit to data and to set the initial time of infection *c*_5_. This function has the “logistic” form: $F(t)=\frac{1}{1+ \exp \left(-{c}_4\left(t-{c}_5\right)\right)},$ (13) where *c*_4_ determines the rate at which the incidence increases.	

To obtain the risk of dengue for foreign visitors we fitted to the data incidence to the continuous function (12) for the force of infection *λ*(*t*), such that when used in the model (11) reproduces the SINAN incidence data. As mentioned in the Introduction, in Brazil, dengue cases, and many other infectious diseases are compulsorily notified. The number of weekly cases are reported to the official depository system of the Brazilian Ministry of Health (SINAN) database. In this paper it is assumed that all clinical (symptomatic) dengue cases are notified and, therefore, SINAN is assumed to be equal to *λ*(*t*)*S*(*t*). This is the quantity that is reproduced by the model. Of course it is also necessary to estimate, after each outbreak, an initial number of susceptible individuals, *S*(0), in order to obtain the incidence of dengue notification in the next outbreak. This is estimated as follows.

The total population of Rio de Janeiro in 2000 was obtained from the census of National Statistics in Demography Institute of Brazil as 5,857,904 inhabitants. The next national census was carried out in 2010 and the total number of inhabitants increased to 6,317,424 individuals. As explained below, we need the total population in each years, which was obtained by interpolation in between. After 2010, the total population was extrapolated from the two precedents censuses.

In order to obtain the initial conditions *S*(0) for Rio for each year we first projected the expected number of inhabitants and discounted the number of dengue cases reported in the previous year. This was carried out for the years 2000 until 2007 because in this period serotype 3 predominated in Rio. For 2008, the total projected population size was used because a new serotype started circulating (DENV-2), to which all inhabitants were assumed susceptible. For 2009 and 2010 the same procedures of discounting the number of cases in the previous years was applied because DENV-2 circulated along these 3 years. In 2011, serotypes 1 and 4 were introduced and the initial condition assumed for simulating the years 2011–2015 were obtained first, by doubling the projected population for 2011 (there were 2 virus circulating) and for 2012–2015 by discounting the number of cases reported in the previous years.

One important aspect to be considered, however, is the expected number of asymptomatic dengue infections, which has been subject of some publications recently. The ratio asymptomatic/symptomatic varies from 2 to 10 according to references [23] and [24]. In addition, there is now evidence suggesting that those asymptomatic individuals are equally or even more transmissible to the mosquitoes [25]. Therefore, we simulated 2 scenarios with the ratios symptomatic/asymptomatic as equal to 1:1 and 1:4.

Table 6 summarize the above procedures.

**Table 6:** Number of susceptible individuals in each year analyzed according to the assumed proportion of symptomatic/asymptomatic

				S_0_ Symptomatic	S_0_ asymptomatic
Year	Virus	Population	Number of reported cases	1:1	1:4
2000	3	5,857,904	4387	5,857,904	5,857,904
2001	3	5,860,374	29607	5,855,987	5,842,826
2002	3	5,906,079	152687	5,872,085	5,770,103
2003	3	5,951,784	3781	5,765,103	5,205,060
2004	3	5,997,489	2606	5,807,027	5,235,641
2005	3	6,043,194	2874	5,850,126	5,270,922
2006	3	6,088,899	16623	5,892,957	5,305,131
2007	3	6,093,472	27340	5,880,907	5,243,212
2008	2	6,180,309	130876	6,180,309	6,180,309
2009	2	6,226,014	5269	6,095,138	5,702,510
2010	2	6,320,446	5477	6,184,301	5,775,866
2011	1 & 4	6,317,424	83357	12,634,848	12,634,848
2012	1 & 4	6,363,129	140559	12,642,901	12,392,830
2013	1 & 4	6,408,834	70077	12,593,752	11,922,004
2014	1 & 4	6,453,682	5699	12,613,371	11,731,392
2015	1 & 4	6,500,244	17504	12,700,796	11,801,720

In the table the 3rd column is the calculated size of the population of Rio in each year. The 4th column is the number of reported (symptomatic) cases. The 5th column is the initial number of susceptibles, *S*(0), calculated by subtracting the total population the number of reported cases considering that there was no asymptomatic cases. Column 6th recalculated *S*(0) by considering that the ratio symptomatic/asymptomatic was 1:4.

For instance, for 2001 we considered *S*(0) as the total population of 2000 minus the number of reported cases in the previous year for the ratio 1:1, minus 2 times the number of reported cases in 2000, and so on for the other ratios considered.

**Fig. 3:**
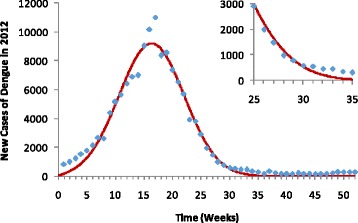
The incidence of dengue in Rio in 2012. The inset box shows the weekly number of new cases reported between the epidemiological weeks 25 and 35. Blue diamonds represent the actual number of weekly reported dengue cases and the continuous red line the model's outcome

Summarizing, we fitted equations (12) and (13) to obtain the parameters *c*_*i*_(*i* = 1,...5) that, when applied to the set of equations (11), assuming as initial conditions for the number of susceptible individuals, *S*(0), as explained above, retrieves the incidence of new cases reported per week in the SINAN (*λ*(*t*)*S*(*t*)) database. Figure 3 shows the quality of the fitting procedure for 2012, the worst year in terms of dengue incidence in Rio de Janeiro for the case were no asymptomatic infections was considered.

## Results

The results of the above calculations are summarized in Tables 7 and 8, which shows the individual risks of infection for travelers and the expected number of cases for each one of the scenarios simulated, respectively.

**Table 7:** Individual risk of acquiring dengue and the respective 95 % Confidence Interval for both scenarios simulated

Year	Individual Risk - symptomatic infection	95 % CI	Individual Risk - asymptomatic infection x4	95 % CI
2000	3.21E-06	[1.73E-06, 4.68E-06]	1.29E-05	[9.90E-06, 1.58E-05]
2001	1.05E-05	[7.87E-06, 1.32E-05]	4.18E-05	[3.65E-05, 4.72E-05]
2002	1.40E-09	[0.00E + 00, 3.22E-08]	7.50E-09	[0.00E + 00, 7.95E-08]
2003	1.58E-07	[0.00E + 00, 4.89E-07]	4.04E-06	[2.28E-06, 5.80E-06]
2004	5.42E-07	[0.00E + 00, 1.15E-06]	2.42E-06	[1.06E-06, 3.78E-06]
2005	2.75E-06	[1.37E-06, 4.12E-06]	1.21E-05	[9.08E-06, 1.51E-05]
2006	2.39E-06	[1.12E-06, 3.67E-06]	1.07E-05	[7.90E-06, 1.36E-05]
2007	5.84E-05	[5.21E-05, 6.47E-05]	5.14E-04	[4.94E-04, 5.34E-04]
2008	4.44E-06	[2.75E-06, 6.14E-06]	3.62E-05	[3.14E-05, 4.11E-05]
2009	2.15E-07	[0.00E + 00, 5.89E-07]	6.83E-06	[4.65E-06, 9.02E-06]
2010	2.39E-05	[1.99E-05, 2.78E-05]	1.09E-04	[1.00E-04, 1.17E-04]
2011	4.45E-06	[3.27E-06, 5.64E-06]	1.82E-05	[1.58E-05, 2.06E-05]
2012	3.75E-05	[3.41E-05, 4.10E-05]	1.62E-04	[1.54E-04, 1.69E-04]
2013	4.18E-07	[5.46E-08, 7.82E-07]	1.82E-06	[1.04E-06, 2.60E-06]
2014	9.25E-06	[7.54E-06, 1.10E-05]	4.01E-05	[3.64E-05, 4.38E-05]
2015	3.72E-05	[3.38E-05, 4.07E-05]	1.61E-04	[1.54E-04, 1.69E-04]

**Table 8:** Expected number of symptomatic and asymptomatic dengue and respective 95 % Confidence Interval for both scenarios simulated

Year	Expected Symptmatic cases	95 % C.I.	Expected Asymptmatic cases	95 % C.I.
2000	1	(1–2)	5	(4–6)
2001	4	(3–5)	17	(15–19)
2002	0	–	0	–
2003	0	–	2	(1–2)
2004	0	–	1	(0–2)
2005	1	(1–2)	5	(4–6)
2006	1	(0–1)	4	(3–5)
2007	23	(21–26)	206	(198–214)
2008	2	(1–2)	14	(13–16)
2009	0	–	3	(2–4)
2010	10	(8–11)	43	(40–47)
2011	2	(1–2)	7	(6–8)
2012	15	(14–16)	65	(62–68)
2013	0	–	1	(0–1)
2014	4	(3–4)	16	(15–18)
2015	15	(14–16)	64	(62–68)

## Discussion

In this paper we propose a model for estimating the individual risk of dengue infection for non-immune visitors attending the 2016 Summer Olympic Games in Rio de Janeiro, Brazil. An SIR model is used for describing the spread of dengue among local inhabitants and a stochastic simplification, assuming an SI structure, was applied to estimate the individual risk of dengue for travelers. The force of infection for the period of the games was calculated by assuming a Gaussian-type function that was fitted to the actual dengue notification system in the country. The maximum risk for travelers, assuming an incidence at the time of the 2016 games as equal to worst month of August (2007) in terms of dengue in Rio, was calculated to be 5.84x10^-5^ (95 % CI: 5.21x10^−5^−6.47x10^−5^) and 5.14x10^−4^ (95 % CI: 4.94x10^−4^−5.34x10^−4^) for a 1:1 and 1:4 symptomatic/asymptomatic ratios, respectively. The fact that the worst month of August occurred in 2007, a year with a relatively low number of dengue cases deserves an explanation. Although the worst dengue year in the historical set studied was 2012, in 2007, the month of August showed a high remaining number of dengue cases and the following dengue season had already started. This was probably due to climatic factors because the average maximum, mean and minimum temperatures recorded for the month of August in 2007 were 3 centigrade degrees higher than 2012 (29, 23 and 20 versus 26, 20 and 18, respectively). This may have result in a higher density of mosquitoes and consequently higher intensity of transmission in that particular year.

This individual risk when scaled to the expected 400,000 non-immune visitors would translate into the expected number of visitors who would acquire a dengue infection during the Olympic Games.

The model relies on several assumptions, namely, that visitors are subject to the same force of infection as the local residents; the visitors are in relatively low number with respect to the local population; visitors spend a relatively short period in Rio de Janeiro and, therefore, do not contribute to the local force of infection; and, finally, there is no competitive risk for travelers, like returning home before acquiring the infection.

We would like to emphasize that our main result is the individual probability that a non-immune traveller acquire the infection. With this probability any individual may decide whether he/she will come to Rio. This individual probability of infection (Table 7) allows the calculation of having any number of travellers *N* will result in *n ≤ N* infections.

To estimate the expected number of travellers that get dengue we explicitly assume (including the title) that all visitors are non-immune. This produces an upper bound to the average number of expected cases/infections.

With respect to immune visitors from dengue endemic countries, we have the following observations:

1. those individuals must come from South-East Asia, Africa or South America, the endemic regions of dengue;
2. judging from the number of visitors attending the World Cup in 2014, from the 700,000 foreign visitors, about half came from South America. The remaining came either from Europe or North America. African and South-Eastern Asians were a small minority, in some cases only represented by the athletes themselves.

Therefore, the expected number of cases/infections could be cut by about half.

Unfortunately, all the four serotypes of dengue are circulating in South American countries and the proportion of immune individuals is unknown and in some cases subject to errors. Therefore, we consider only 400,000 that are roughly the estimation of non-immune visitors.

The main shortcoming with epidemiological forecasting is to establish the extent to which the past is likely to be an accurate guide to the future [26]. There are many unpredictable factors that generate uncertainties at many levels, and often policy makers have to rely on information of previous outbreaks in order to make decisions that are at high stake, both in terms of health and economics. Woolhouse proposes 4 scenarios for forecasting. (1) Data and values of parameters for previous outbreaks are assumed to be valid for future episodes and the structure of the model is assumed to be valid as well, in other words there is no change; (2) There may be changes in the dengue serotype circulating at the moment of the forecasting, or in the intensity of transmission, or in climate condition for the breeding of mosquitoes, in other words the input data change; (3) The parameter values change, such as the force of infection of dengue as determined by the density of infected mosquitoes with respect to human hosts and the biting rate. These kinds of changes would be difficult to quantify a priori, and new parameter values may need to be estimated from earlier epidemic data; and (4) the model changes, which means the original model structure or assumptions are incorrect for the new epidemic.

All or some of these factors may explain the failure to accurately predict the number of dengue cases for the World Cup 2014 in Brazil, and the forecasting for the Olympic Games is not immune to them. We show that the year 2014 experienced a particularly low dengue incidence and when we run our model for that year (but after the event of the World Cup) we see that zero to a handful of cases during the World Cup was a likely possibility. Climatic events such as a severe drought in 2014 could explain the low incidence of dengue in that year but other factors could have contributed as well.

The accuracy of any attempt to forecast the number of dengue cases for the Olympic Games in 2016 will very much depend on the intensity of transmission. This in turn is dependent on changes in the serotype circulating, climate conditions and host behavior. Furthermore, the Olympic Games will motivate public health practitioners to intensify vector control measures around the Games, thereby reducing the risk of dengue in the vicinity of the Games. In addition, the oscillatory distribution of cases is of upmost importance for the risk of dengue during the period of time of the Olympic Games. As can be seen in Fig. 1 the years of 2002 and 2008 showed the highest peaks in the number of cases but these were concentrated during the summer season. With the exception of 2007, the month of August in those years showed a very low incidence of dengue and hence the low expected number of cases projected for the Olympics in Table 8. Hence, if we apply the same level of dengue over the last 15 years, the expected number of symptomatic dengue among non-immune visitors attending the Olympic Games in Rio de Janeiro in 2016 will vary depending on the intensity of transmission of dengue in the month of August. This, in turn, will depend on climatic factors and the number of infected mosquitoes in that particular month.

After we finished the revision of the original manuscript Brazil has been overwhelmed by an apparently huge outbreak of Zika virus. This triggered a wave of concern around the world, in particular due to the still to be confirmed association with microcephaly in babies born from mothers infected with Zika. The calculations provided by the present work could represent an important step toward understanding and quantifying the risk of exposure to Zika for travellers visiting Rio during the Olympic Games and eventually bitten by Aedes mosquitoes. Preliminary calculations suggest a very low individual risk on the order of 2x10^-6^ but this is still based on incomplete information. As soon as more information on Zika incidence in Brazil is known through new and reliable diagnostic methods we intend to calculate the risk of Zika infection for travellers visiting Brazil any time in the future

## Conclusions

If dengue returns in 2016 with the pattern observed in the worst month of August in history (2007), the expected number of symptomatic and asymptomatic dengue cases among tourists will be 23 and 206 cases, respectively. This worst case scenario would have an incidence of 5.75 (symptomatic) and 51.5 (asymptomatic) per 100,000 individuals.

Preliminary calculations on the risk of Zika virus infection for tourists suggest a very low individual risk on the order of 2x10^−6^ but this is still based on incomplete information.

### Ethics approval and consent to participate

This work has been approved by the Ethics Review Board of the School of Medicine of the University of Sao Paulo. No human subjects were involved.

### Availability of data and materials

All data used has been retrieved from the SINAN system, available at: http://portalsaude.saude.gov.br/index.php/oministerio/principal/secretarias/svs/dengue

## Abbreviations

DENV: Dengue virus
DHF: Dengue hemorragic fever
FIFA: “Fédération Internationale de Football Association”
LIM: “Laboratórios de Investigação Médica”
PGF: Probability generating function
SI: Susceptible - infected
SINAN: “Sistema de Informação de Agravos de Notificação”
SIR: Susceptible-infected-removed

## References

[1] Khan K, Freifeld CC, Wang J (2010). Preparing for infectious disease threats at mass gatherings: the case of the Vancouver 2010 Olympic Winter Games. CMAJ.

[2] Murray NE, Quam MB, Wilder-Smith A (2013). Epidemiology of dengue: past, present and future prospects. Clin Epidemiol.

[3] Teixeira MG, Costa Mda C, Barreto F, Barreto ML (2009). Dengue: twenty-five years since reemergence in Brazil. Cad Saude Publica.

[4] Teixeira MG, Siqueira JB, Ferreira GL, Bricks L, Joint G (2013). Epidemiological trends of dengue disease in Brazil (2000-2010): a systematic literature search and analysis. PLoS Negl Trop Dis.

[5] Wilson ME, Chen LH, Han PV (2014). Illness in travelers returned from Brazil: the GeoSentinel experience and implications for the 2014 FIFA World Cup and the 2016 Summer Olympics. Clin Infect Dis.

[6] Aguiar M, Coelho GE, Rocha F, Mateus L, Pessanha JE, Stollenwerk N (2015). Dengue transmission during the 2014 FIFA World Cup in Brazil. Lancet Infect Dis.

[7] Massad E, Wilder-Smith A, Ximenes R (2014). Risk of symptomatic dengue for foreign visitors to the 2014 FIFA World Cup in Brazil. Mem Inst Oswaldo Cruz.

[8] Aguiar M, Rocha F, Pessanha JE, Mateus L, Stollenwerk N (2015). Corrigendum: Carnival or football, is there a real risk for acquiring dengue fever in Brazil during holidays seasons?. Sci Rep.

[9] Barcellos C, Lowe R (2014). Dengue and the world football cup: a matter of timing. PLoS Negl Trop Dis.

[10] Harley D, Viennet E (2014). Football fans and fevers: dengue and the World Cup in Brazil. Lancet Infect Dis.

[11] Hay S (2013). Football fever could be a dose of dengue. Nature.

[12] Lowe R, Barcellos C, Coelho CA (2014). Dengue outlook for the World Cup in Brazil: an early warning model framework driven by real-time seasonal climate forecasts. Lancet Infect Dis.

[13] Coordenação Geral do Programa Nacional de Controle da Dengue. Ministry of Health Brazil. Available at: http://portalsaude.saude.gov.br/index.php/oministerio/principal/secretarias/svs/dengue Accessed in 3 July 2015

[14] Earnest A, Tan SB, Wilder-Smith A (2012). Meteorological factors and El Nino Southern Oscillation are independently associated with dengue infections. Epidemiol Infect.

[15] Liu-Helmersson J, Stenlund H, Wilder-Smith A, Rocklov J (2014). Vectorial capacity of *Aedes aegyptii*: effects of temperature and implications for global dengue epidemic potential. PLoS One.

[16] Wilder-Smith A, Ooi EE, Vasudevan SG, Gubler DJ (2010). Update on dengue: epidemiology, virus evolution, antiviral drugs, and vaccine development. Curr Infect Dis Rep.

[17] Nogueira RM, de Araujo JM, Schatzmayr HG (2007). Dengue viruses in Brazil, 1986-2006. Rev Panam Salud Publica.

[18] Rodriguez-Barraquer I, Cordeiro MT, Braga C, de Souza WV, Marques ET, Cummings DA (2011). From re-emergence to hyperendemicity: the natural history of the dengue epidemic in Brazil. PLoS Negl Trop Dis.

[19] Heringer M, Nogueira RM, de Filippis AM (2015). Impact of the emergence and re-emergence of different dengue viruses’ serotypes in Rio de Janeiro, Brazil, 2010 to 2012. Trans R Soc Trop Med Hyg.

[20] Massad E, Coutinho FAB, Yang HM, de Carvalho HB, Mesquita R, Burattini MN (1994). The basic reproduction ration of HIV among intravenous drug-users. Math Biosc.

[21] Amaku M, Azevedo F, Burattini MN, Coutinho FAB, Lopez LF, Massad E (2015). Interpretations and pitfalls in modelling vector-trasmitted infections. Epidemiol Infect.

[22] Coelho GE, Burattini MN, Teixeira MD, Coutinho FAB, Massad E (2008). Dynamics of the 2006/2007 dengue outbreak in Brazil. Mem Inst Oswaldo Cruz.

[23] Chastel C (2012). Eventual role of asymptomatic cases of dengue for the entroduction and spread of dengue viruses in non-epidemic regions. Front Physiol.

[24] Bhatt S, Gething PW, Brady OJ, Messina JP, Farlow AW, Moyes CL, Drake JM, Brownstein JS, Hoen AG, Sankoh O, Myers MF, George DB, Jaenisch T, Wint GR, Simmons CP, Scott TW, Farrar JJ, Hay SI (2012). The global distribution and burden of dengue. Nature.

[25] Duong V, Lambrechts L, Paul RE, Ly S, Lay RS, Long KC, Huy R, Tarantola A, Scott TW, Sakuntabhai AJ, Buchy P (2015). Asymptomatic humans transmit dengue virus to mosquitoes. PNAS.

[26] Woolhouse M (2011). How to make predictions about future infectious disease risks. Philos Trans R Soc Lond B Biol Sci.

